# Gut-engineered *Bacillus* subtilis-mediated BAMBI delivery for the treatment of thioacetamide-induced liver fibrosis through mechanotransduction inhibition

**DOI:** 10.3389/fbioe.2026.1817519

**Published:** 2026-05-08

**Authors:** Xiaochang Zhao, Yu Zhang, Xiangyu Chu, Wenqian Ma, Rongkai Zhan, Yan Wang, Haiyan Fang, Peng Guo, Ziye Wang, Xiaohan Chen, Jiapeng Yang, Xiaoxia Sun

**Affiliations:** 1 Shandong Institute of Medical Device and Pharmaceutical Packaging Inspection, Jinan, Shandong, China; 2 National Laboratory of Solid State Microstructures, Department of Physics, Nanjing University, Nanjing, China; 3 Jinan Microecological Biomedicine Shandong Laboratory, Jinan, China; 4 MOE Key Laboratory of High Performance Polymer Materials and Technology, Chemistry and Biomedicine Innovation Center (ChemBIC), School of Chemistry, Nanjing University, Nanjing, China; 5 School of Chemistry and Chemical Engineering, Nanchang University, Nanchang, China; 6 Department of Chemical and Biomolecular Engineering, National University of Singapore, Singapore, Singapore

**Keywords:** BAMBI, extracellular matrix remodeling, gut-liver axis, liver fibrosis therapy, mechanotransduction, TGF-β signaling

## Abstract

Liver fibrosis, driven by chronic injury and excessive extracellular matrix (ECM) deposition, lacks effective clinical therapies. This study pioneers a strategy employing genetically engineered *Bacillus subtilis* (strain Bs-BAMBI-8) to constitutively secrete bone morphogenetic protein and activin membrane-bound inhibitor (BAMBI), which is subsequently delivered to the liver via the gut-liver axis to therapeutically antagonize liver fibrosis. Methodologically, liver fibrosis was induced in mice via 6-week intraperitoneal injections of thioacetamide (TAA), followed by a 19-week daily oral gavage of live Bs-BAMBI-8 (10^9^ CFU). This longitudinal intervention achieved sustained intestinal colonization (>10^5^ CFU) and BAMBI translocation via the gut-liver axis. This intervention significantly reduced hepatic fibrosis, evidenced by decreased NAFLD Activity Score from six to four and regression of fibrosis stage from S3 to S2. Mechanistically, BAMBI acted as a decoy receptor for transforming growth factor-beta (TGF-β), inhibiting TGF-β signaling and downregulating fibrotic markers (α-smooth muscle actin, collagen I, phosphorylated focal adhesion kinase). This suppression disrupted ECM-mediated mechanotransduction pathways, attenuating hepatic stellate cell activation. Concomitantly, serum markers (alanine aminotransferase, aspartate aminotransferase, alkaline phosphatase, total bilirubin) recovered, while albumin synthesis and platelet count recovered. Crucially, this engineered microbiome-based approach integrates synthetic biology with mechanobiology to simultaneously target biochemical signaling and mechanical transduction. It establishes a potentially translatable paradigm for chronic liver disease therapy.

## 1 Introduction

Liver fibrosis (LF) is a critical pathological process induced by chronic liver injury, characterized by excessive accumulation of extracellular matrix (ECM) proteins (Parola and Pinzani, 2024; Bataller and Brenner, 2005; Bogenschutz et al., 2020). As fibrosis progresses, liver structure and function are progressively compromised, ultimately leading to cirrhosis and liver failure (Bataller and Brenner, 2005; Seki and Schwabe, 2015; Tsuchida and Friedman, 2017; Zhang et al., 2023; Zhao et al., 2016). LF represents a significant global health issue, particularly among patients with non-alcoholic fatty liver disease (NAFLD), viral hepatitis, and other chronic liver diseases (Schuppan and Afdhal, 2008; Rinella et al., 2019; Lyu et al., 2023). Currently, therapeutic options for advanced liver fibrosis are limited, and once fibrosis progresses to cirrhosis, no effective drugs are available to reverse the damage. Therefore, there is an urgent need for the development of novel, targeted strategies to manage and reverse liver fibrosis (Paul et al., 2022; Marra and Tacke, 2014; Albillos et al., 2020; Paquissi, 2017; Weber et al., 2023).

In recent years, the role of the gut microbiome in host liver diseases has garnered increasing attention. The gut-liver axis, a bidirectional communication pathway mediated through the portal vein, plays a pivotal role in liver pathophysiology (Schuppan and Afdhal, 2008; Wang et al., 2017; Wahlström et al., 2016; Sarashina-Kida et al., 2017; D'Aldebert et al., 2009). Signals and metabolites derived from the gut microbiome can directly influence liver function and immune responses (Albillos et al., 2020; Triger et al., 1978; Tarao et al., 1977). This discovery has provided a new perspective for the treatment of liver diseases (Marcellin et al., 2013; Guilliams et al., 2022). Engineering the gut microbiome to regulate liver diseases, especially liver fibrosis, has emerged as a promising research direction. Genetically engineered gut bacteria can be designed to colonize the intestine and secrete therapeutic molecules, thereby modulating signaling pathways involved in liver fibrosis (Trebicka et al., 2021a; Ladenheim et al., 1995).

While traditional microbiome interventions focus on broad ecological restoration, emerging research on bacteria-derived proteases has shown direct anti-fibrotic potential in thioacetamide (TAA) models (Albillos et al., 2020); our study bridges the remaining gap by utilizing engineered microbes as 'living factories’ for targeted delivery via the portal vein (Cubillos-Ruiz et al., 2021) to constitutively secrete bone morphogenetic protein and activin membrane-bound inhibitor (BAMBI), providing a dual-action strategy that both inhibits the TGF-β type I receptor (TGF-β) biochemical signaling and disrupts the mechanical feedback loop of ECM remodeling.

Among these emerging strategies, BAMBI, a decoy receptor, has attracted significant attention for its role in the treatment of liver fibrosis (Luedde and Trautwein, 2008; Friedman, 2007). BAMBI shares structural similarities with the extracellular domain of TGF-β and acts by binding to TGF-β, disrupting the formation of receptor complexes and inhibiting the signaling of TGF-β and Bone Morphogenetic Protein (BMP) ligands (Onichtchouk et al., 1999). TGF-β signaling is widely regarded as a core driver of hepatic stellate cell (HSC) activation, a key event in the progression of liver fibrosis. Activated HSCs transdifferentiate into myofibroblasts, express α-smooth muscle actin (α-SMA), and secrete excessive ECM proteins, which continue to promote fibrosis through ECM accumulation and mechanotransduction pathways (Xu et al., 2016; Martinez-Hackert et al., 2021; Huang and Chen, 2012; Trebicka et al., 2021b). Critically, recent mechanobiology studies utilizing photoresponsive hydrogels demonstrate that rapid cyclic rigidity changes dynamically accumulate mechanosignaling proteins (e.g., phosphorylated focal adhesion kinase [pFAK], phosphorylated myosin IIa [pMyosin IIa]) within hepatic stellate cells, amplifying their traction forces and accelerating fibrotic matrix remodeling - establishing a biomechanical amplification loop that drives fibrosis progression (Yang et al., 2025a; Yang et al., 2025b).

The innovation of this study lies in the use of engineered gut bacteria to express BAMBI protein, which is then transported to the liver via the gut-liver axis (Ladenheim et al., 1995). These engineered bacteria are designed to stably colonize the mice intestine and secrete BAMBI, inhibiting the TGF-β signaling pathway and effectively reducing HSC activation (Marco et al., 2018; Herrera et al., 2018). We hypothesize that BAMBI, as a decoy receptor, interferes with the transmission of mechanical and biological signals within the liver fibrosis process by binding to TGF-β, thus reversing the progression of fibrosis. This approach combines biological and physical interventions, disrupting mechanical signal transmission in a manner that biologically reverses the liver fibrosis process. Our research provides a new framework for developing minimally invasive, targeted therapeutic interventions for liver fibrosis, laying the foundation for advancing chronic liver disease management through engineered microbiomes.

## 2 Materials and methods

### 2.1 Construction and screening of the BAMBI expression vector

The recombinant plasmid for secretory expression of human BAMBI protein was constructed using the pBE-S shuttle vector system. The BAMBI coding sequence (Supplementary Table S1) was initially cloned into the multiple cloning site downstream of the native aprE promoter and signal peptide. To screen for optimal secretion efficiency, the aprE signal peptide was replaced with a library of 173 *Bacillus* subtilis-derived secretory signals via In-Fusion HD cloning, where the recombinant vector was first linearized by Mlu I/Eco52 I digestion, ligated with the SP DNA mixture, and transformed into *E. coli* HST08 competent cells. The resulting plasmid library (>2,000 colonies) was pooled and purified prior to transformation into the protease-deficient *B. subtilis* strain RIK1285 using EGTA-assisted competence induction, with transformants subsequently selected on Luria-Bertani (LB) agar plates containing 10 μg/mL kanamycin (McDonald et al., 1995). To quantify the secretion efficiency, the culture supernatants of the transformants were collected after 24 h of incubation. The concentration of secreted BAMBI was measured using a ElaBoX^TM^Human BAMBI ELISA Kit (Solarbio) according to the manufacturer’s instructions. The optical density (OD) was measured at 450 nm using a microplate reader, and the absolute BAMBI concentrations were calculated based on a standard curve.

### 2.2 Animal studies and ethical considerations

All animal experimental protocols were approved by the Institutional Animal Care and Use Committee (IACUC). Wild-type KM mice (6–8 weeks old) were randomly divided into three groups: Control group, LF group, and LF + BAMBI group. All mice received intraperitoneal interventions for 6 weeks: Control (Saline), LF and LF + BAMBI (TAA, 150 mg/kg/day), All mice were fed standard chow with daily water replacement (Yeh et al., 2004; Wallace et al., 2015). Immediately following successful 6-week fibrotic modeling, all groups received daily oral gavage for 19 consecutive weeks with same volume (2 mL per 20 g mice): both control and model groups were administered saline, while the LF + BAMBI group received live *Bacillus subtilis* Bs-BAMBI-8 (10^9^ colony-forming units [CFU] in saline). At 2-week intervals (weeks 1, 3, 5, 7, 9, 11, 13, 15, 17, and 19), five mice per group were euthanized for blood collection via retro-orbital puncture, liver sampling (portions were stored at −80 °C or fixed in 4% paraformaldehyde for ≥48 h), and preservation of intestinal contents (cecum, colon, rectum) and fecal samples (−80 °C). Fresh liver tissues were homogenized in liquid nitrogen for total protein extraction, followed by BAMBI quantification via Western blotting. Fixed liver tissues were paraffin-embedded and sectioned for histopathological analysis. Intestinal contents and fecal samples were cultured on LB agar supplemented with 10 μg/mL kanamycin to isolate antibiotic-resistant colonies. To confirm the successful colonization of Bs-BAMBI-8, colony PCR was performed using specific primers (sequences are provided in Supplementary Table S1), and the resulting amplicons were verified by agarose gel electrophoresis. All experiments were independently replicated five times (n = 5), and data are expressed as mean ± standard deviation.

### 2.3 Blood routine and biochemical assessment

At 1, 3, 5, 7, 9, 11, 13-, 15-, 17-, and 19-week post-gavage (2-week intervals), blood samples were collected via ocular puncture (n = 5 per group per time point). Whole blood was immediately analyzed using an automated hematology analyzer to determine, platelet count (PLT). Serum was separated for quantification of hepatic function markers—alanine aminotransferase (ALT), aspartate aminotransferase (AST), alkaline phosphatase (ALP), albumin (ALB), total bilirubin (TBIL) using a biochemical autoanalyze, following manufacturer protocols. All assays were independently replicated five times (n = 5) (Wu et al., 2024; Mohamed et al., 2025; Ezhilarasan, 2023).

### 2.4 Histological analysis

Liver tissues were fixed in 4% paraformaldehyde for ≥48 h, followed by paraffin embedding. Serial sections (6 μm thickness) were prepared using a Leica SM 2000R microtome. Hematoxylin-eosin (HE) and Masson’s trichrome staining were performed according to the manufacturer’s protocols (Solarbio, Beijing, China) (Wen et al., 2022; Lan et al., 2024; Koda et al., 2021).

To ensure objectivity, the stained slides were evaluated by two independent, experienced pathologists in a blinded manner. General liver injury and inflammation were semi-quantitatively assessed using the NAFLD Activity Score (NAS). The degree of liver fibrosis was evaluated based on Masson’s trichrome staining and graded from stage S0 to S4 according to established histological staging criteria. Histological analysis was conducted using the NAS system: steatosis (0–3), ballooning degeneration (0–2), inflammation (0–2), and fibrosis (0–4) (Kleiner et al., 2005; Brunt, 2001). Fibrosis staging adhered to the European Steatosis, Activity, and Fibrosis (SAF), with F1 stage subclassified as F1a (periportal fibrosis), F1b (focal pericellular fibrosis), and F1c (extensive pericellular fibrosis) (Bedossa et al., 2012).

### 2.5 Cell culture assay

NIH/3T3 fibroblasts were cultured in Dulbecco’s Modified Eagle Medium (DMEM) supplemented with 10% fetal bovine serum and 1% penicillin/streptomycin at 37 °C in a humidified atmosphere containing 5% CO_2_. The culture medium was replaced every two to 3 days, and cells were passaged upon reaching approximately 90% confluency. For experiments, cells were seeded onto the substrate at a density of 1 × 10^6^ cells/mL and allowed to adhere and proliferate for over 24 h under standard culture conditions prior to any experimental procedures. No specific pre-treatments, such as light stimulation, were applied during this initial culture period.

### 2.6 Immunofluorescence staining

Cells were fixed with 4% paraformaldehyde for 15 min at room temperature. After fixation, cells were permeabilized with 0.25% (v/v) Triton X-100 in PBS for 10 min. Non-specific binding sites were blocked by incubation with 1% (w/v) bovine serum albumin (BSA) in PBST for 1 h. The samples were then incubated overnight at 4 °C with the appropriate primary antibodies diluted in the blocking solution. After washing, the cells were incubated with fluorophore-conjugated secondary antibodies for 1 h at room temperature. Cell nuclei were counterstained with DAPI. Fluorescence images were captured using a confocal microscope.

### 2.7 Western blot analysis

Treated cells were washed with cold PBS and lysed on ice using RIPA lysis buffer supplemented with protease inhibitors. Cell lysates were centrifuged at 12,000 × g for 15 min at 4 °C to remove cellular debris. The protein concentration of the supernatant was determined using a bicinchoninic acid (BCA) assay. Equal amounts of protein from each sample were separated by SDS-polyacrylamide gel electrophoresis (SDS-PAGE) and transferred onto polyvinylidene difluoride (PVDF) membranes. The membranes were blocked with 5% non-fat dry milk in TBST for 1 h at room temperature and subsequently incubated overnight at 4 °C with the relevant primary antibodies. After washing, the membranes were incubated with horseradish peroxidase (HRP)-conjugated secondary antibodies for 1 h at room temperature. Protein bands were visualized using an enhanced chemiluminescence (ECL) substrate and detected with a chemiluminescence imaging system. Quantitative densitometric analysis of the immunoblot images was performed using ImageJ software to calculate the relative protein expression levels.

### 2.8 Statistical analysis

All quantitative data are expressed as the mean ± standard deviation (SD). For *in vivo* studies, the sample size was strictly defined as n = 5 biological replicates per group at each specific experimental time point. Because different cohorts of mice were euthanized at each time point, all samples were strictly independent, and no repeated-measures designs were involved. Statistical analyses were performed using GraphPad Prism 9 (GraphPad Software, San Diego, CA). Differences between two independent groups (e.g., comparing the same experimental group between two different time points) were evaluated using a two-tailed, unpaired Student’s t-test. For comparisons among three groups at a single time point, a one-way analysis of variance (ANOVA) followed by Tukey’s *post hoc* test was utilized to correct for multiple comparisons. Exact p-values are explicitly reported in the figure where applicable. Statistical significance was defined as *p < 0.05, **p < 0.01, and ***p < 0.001.

## 3 Results

### 3.1 Engineered *Bacillus subtilis* delivers BAMBI via gut-liver axis to ameliorate liver fibrosis

**FIGURE 1:**
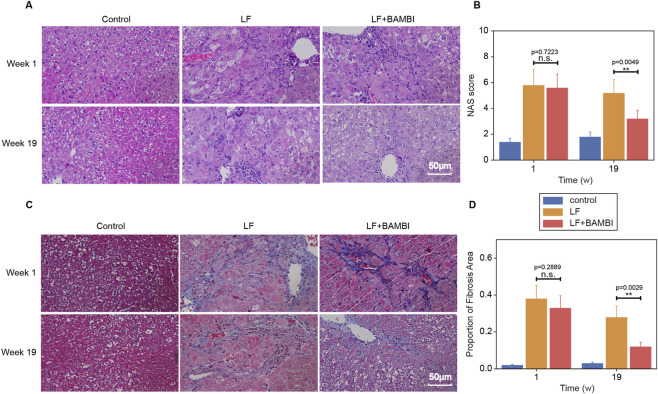
BAMBI Drives Hepatic Repair Through Injury Suppression and Fibrosis Resolution. **(A,B)** HE staining showed reduced ballooning degeneration and inflammatory infiltration in the LF + BAMBI group at week 19 versus persistent damage in the LF group. **(C,D)** Longitudinal Masson staining (weeks 1–19) revealed diminished collagen deposition and pseudolobule formation in LF + BAMBI by week 19, with fibrosis stage declining from S3 to S2. Scale bars are 50 μm for all panels. Data expressed as mean ± SD (**p < 0.01, n = 5). The time scale is indicated in weeks (w).

**FIGURE 2:**
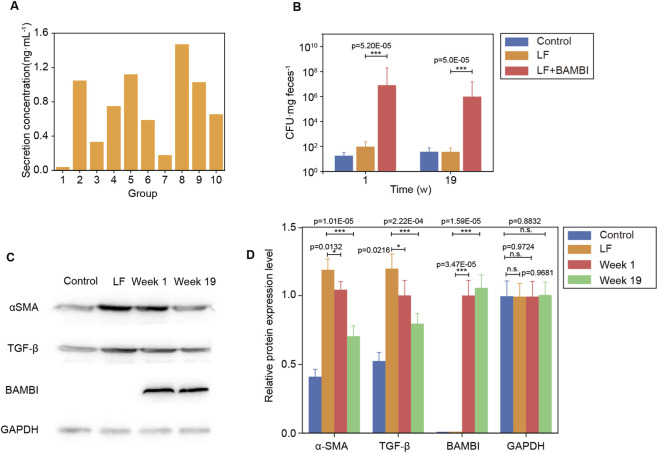
Engineered *Bacillus subtilis* Alleviates Liver Fibrosis via Gut-Liver Axis-Mediated BAMBI Delivery. **(A)** Screening of engineered *B. subtilis* strains by ELISA identified Bs-BAMBI-8 with the highest BAMBI secretion. **(B)** Antibiotic-resistant Bs-BAMBI-8 showed significantly enhanced intestinal colonization in the LF + BAMBI group compared to the control and LF groups. **(C)** Representative Western blot images showing the hepatic protein expression levels of BAMBI, α-SMA, and TGF-β. **(D)** Quantitative densitometric analysis of BAMBI, α-SMA, and TGF-β protein levels normalized to GAPDH. The quantitative results demonstrate that the LF + BAMBI group exhibited significantly upregulated BAMBI expression, alongside suppressed α-SMA, and TGF-β expression, compared to the untreated LF group. Data are expressed as mean ± SD (*p < 0.05, **p < 0.01, ***p < 0.001). The time scale is indicated in weeks (w).

A LF model was successfully established in 6- to 8-week-old KM mice via a 6-week course of intraperitoneal TAA injections (Supplementary Figure S1). Histopathological analysis (HE and Masson staining) revealed that TAA-treated mice exhibited darkened liver coloration, disrupted lobular architecture, accompanied by lipid accumulation, inflammatory infiltration, and collagen deposition (NAS = 6, fibrosis stage S3) (Figure 1). Following 19 weeks of oral intervention with the genetically engineered *B. subtilis* strain Bs-BAMBI-8 (selected for highest BAMBI secretion via ELISA screening, Figure 2A), the LF + BAMBI group demonstrated significantly enhanced intestinal colonization (confirmed by CFU counts under antibiotic selection, Figure 2B and verified as Bs-BAMBI-8 by PCR; Supplementary Figure S2) and upregulated hepatic BAMBI expression (validated by Western blotting and subsequent densitometric quantification, Figures 2C,D), indicating efficient gut-liver axis translocation of the bacterial-derived BAMBI. Mechanistically, Western blotting analysis and quantitative results further showed downregulated expression levels of α-SMA and TGF-β in the liver (Figures 2C,D), suggesting that Bs-BAMBI suppresses the TGF-β signaling pathway by overexpressing BAMBI, thereby reducing α-SMA and TGF-β expression and alleviating fibrotic progression. The blots with molecular weight markers were shown in Supplementary Figure S3. This intervention reduced the NAS to four and improved fibrosis staging to S2, demonstrating effective reversal of hepatic fibrosis (Figure 1).

### 3.2 BAMBI promotes hepatic repair by suppressing injury and fibrotic remodeling

HE staining showed significantly attenuated hepatic pathological damage in the LF + BAMBI group (successfully colonized with engineered bacteria) compared to the untreated group (Figure 1A). At week one of intervention, both the LF and LF + BAMBI groups still exhibited elevated levels of hepatocyte edema, apoptosis, and inflammatory infiltration. By week 19, the LF + BAMBI group demonstrated near-complete resolution of ballooning degeneration and necrosis, along with reduced inflammatory responses. Masson’s trichrome staining (weeks 1–19) revealed that bridging fibrosis and pseudolobule formation were present in both groups at week 1. However, by week 19, the LF + BAMBI group showed significantly reduced collagen deposition, with quantitative analysis of fibrotic area via image analysis software confirming a marked decrease in fibrosis severity (Figures 1C,D), and fibrosis stage improved from S3 to S2. These findings demonstrate that 19 weeks of intervention significantly reduced hepatic inflammation and fibrosis, highlighting BAMBI’s dual mechanism of action—simultaneously suppressing inflammation and promoting fibrotic resolution, thereby facilitating tissue recovery and supporting its therapeutic potential for chronic liver diseases.

### 3.3 BAMBI orchestrates temporal restoration of hepatic homeostasis and inflammatory resolution

**FIGURE 3:**
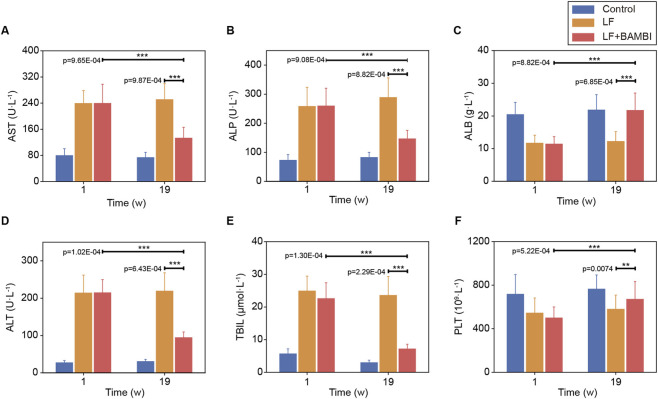
Serum biochemistry and hematological parameters in mice after BAMBI administration. **(A)** AST levels at 1 week and 19 weeks post-administration. **(B)** ALP levels at 1 week and 19 weeks post-administration. **(C)** ALB levels at 1 week and 19 weeks post-administration. **(D)** ALTlevels at 1 week and 19 weeks post-administration. **(E)** TBIL levels at 1 week and 19 weeks post-administration. **(F)** PLT at 1 week and 19 weeks post-administration. Data expressed as mean ± SD (*p < 0.05, **p < 0.01, ***p < 0.001, n = 5). The time scale is indicated in weeks (w).

Serum biochemical analysis revealed that at week one post-intervention, the LF + BAMBI group maintained elevated levels of liver injury markers (ALT, AST, ALP, TBIL) alongside reduced ALB synthesis and PLT counts. By week 19, this group exhibited normalized injury markers, enhanced ALB production (Figure 3C), PLT recovery (Figure 3F), and significant reductions in ALT, AST, ALP levels (Figures 3A,B,D), with TBIL (Figure 3E) approaching control levels. These biochemical findings demonstrate BAMBI-mediated suppression of fibrotic progression and subsequent reversal, ultimately promoting functional hepatic restoration.

### 3.4 BAMBI attenuates ECM protein secretion via sustained TGF-β pathway inhibition

**FIGURE 4:**
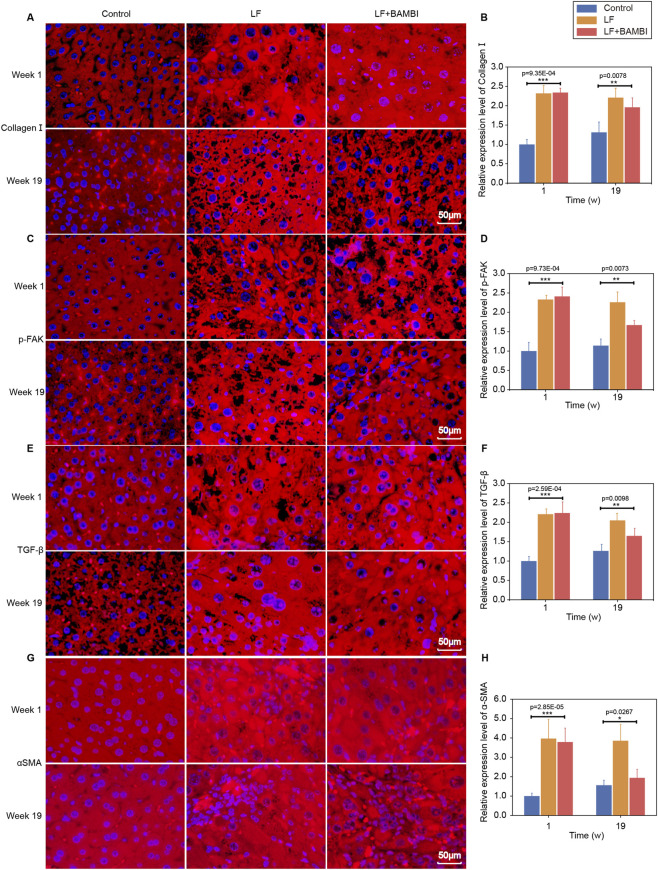
Tissue immunofluorescence analysis of fibrotic markers in BAMBI-treated mice. **(A)** Collagen I staining. **(B)** Semi-quantification of collagen I. **(C)** p-FAK staining. **(D)** Semi-quantitative of p-FAK. **(E)** TGF-β staining. **(F)** Semi-quantification of TGF-β. **(G)** α-SMA staining. **(H)** Semi-quantitative of α-SMA. Data expressed as mean ± SD (*p < 0.05, **p < 0.01, ***p < 0.001, n = 5). Scale bars are 50 μm for all panels. The time scale is indicated in weeks (w).

**FIGURE 5:**
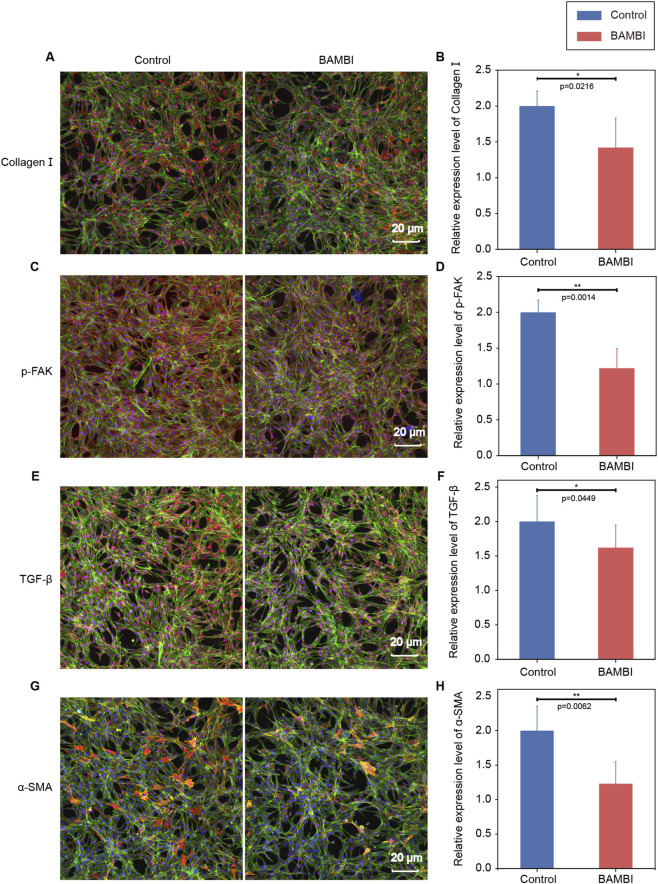
Immunofluorescence analysis of fibrotic markers in cultured fibrotic cells treated with BAMBI. **(A)** Collagen I staining. **(B)** Semi-quantification of collagen I. **(C)** p-FAK staining. **(D)** Semi-quantification of p-FAK. **(E)** TGF-β staining. **(F)** Semi-quantification of TGF-β. **(G)** α-SMA staining. **(H)** Semi-quantitative of α-SMA. Data expressed as mean ± SD (*p < 0.05, **p < 0.01, ***p < 0.001, n = 5). Scale bars are 20 μm for all panels. The time scale is indicated in weeks (w).

Quantitative analysis of relative fluorescence intensity (normalized to week one control group = 1) revealed heightened activation of TGF-β signaling during advanced fibrogenesis: Both LF and LF + BAMBI groups showed increases in fluorescence intensities of collagen I, p-FAK, TGF-β, and α-SMA compared to controls at week 1 (Figure 4). By week 19, the LF + BAMBI group exhibited varying degrees of reduction in these marker intensities versus LF controls, confirming BAMBI’s sustained inhibition of TGF-β signaling (Figure 4). Consistently, in cultured fibroblasts, BAMBI treatment induced notable reductions in fluorescence intensities of all four proteins (Figure 5). These results establish that BAMBI disrupts the fibrogenic activation loop by selectively downregulating TGF-β pathway-driven secretion of ECM-related proteins (collagen I, p-FAK) and autocrine production of TGF-β/α-SMA.

## 4 Discussion

This study establishes a novel therapeutic paradigm for LF by targeting mechanotransduction pathways through gut-engineered *Bacillus subtilis* secreting BAMBI. Our results demonstrate that oral delivery of Bs-BAMBI-8 achieves stable intestinal colonization (>10^5^ CFU, Figure 2B) and hepatic BAMBI translocation, thereby not only halting fibrogenesis but also promoting functional recovery—reducing NAS scores from six to four and reversing fibrosis from stage S3 to S2 (Figures 1–3). Mechanistically, BAMBI acts as a decoy receptor for TGF-β, disrupting TGF-β/Smad signaling—a core driver of HSC activation and ECM deposition. Longitudinal analyses revealed its dual therapeutic action: early-phase blockade of TGF-β-driven injury (stabilized ALT/AST/ALP/TBIL and preserved ALB without altering PLT at week 1, Figure 3), followed by late-phase resolution of fibrotic matrices via reduction in collagen I, p-FAK, TGF-β, and α-SMA (Figure 4). This ECM dissolution spatially correlated with PLT rebound and ALB normalization (week 19), confirming that BAMBI facilitates hepatic regeneration primarily by remodeling the fibrotic niche rather than direct proliferative stimulation.

**FIGURE 6:**
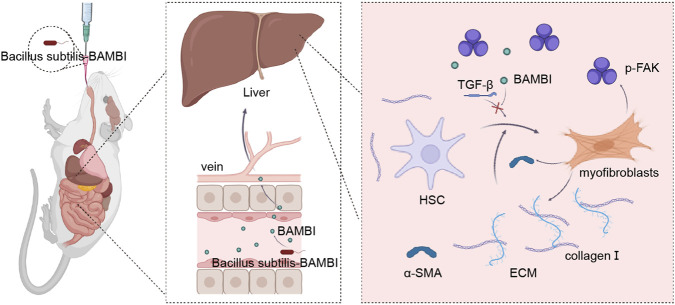
Mechanisms of BAMBI in inhibiting liver fibrosis.

Crucially, our strategy advances beyond conventional anti-fibrotic approaches and recent microbiome-based therapies. Our findings strongly align with previous studies demonstrating that BAMBI overexpression or TGF-β blockade significantly attenuates liver fibrogenesis (Xu et al., 2016; Herrera et al., 2018; Salem et al., 2023). For instance, while it has been recently demonstrated that bacterial proteases can directly alleviate TAA-induced liver fibrosis by suppressing HSC proliferation, our approach offers a distinct advantage by concurrently targeting biochemical signaling (TGF-β inhibition) and mechanical transduction (Salem et al., 2023). Furthermore, when compared to conventional microbiome-based therapies that primarily rely on restoring microbial dysbiosis or modulating general systemic inflammation, our engineered Bs-BAMBI-8 platform provides a targeted mechanistic intervention into the physical microenvironment. BAMBI-mediated interference with TGF-β uniquely disrupts the “ECM stiffness → HSC activation” feedback loop (Figure 6), aligning with emerging evidence that ECM mechanics drive fibrosis progression. The gut-centric delivery platform further enhances translational potential: it bypasses systemic drug limitations (e.g., poor bioavailability, off-target effects) and leverages the microbiome’s endogenous role in liver homeostasis. Notably, the use of TAA for LF modeling (versus toxic CCl_4_) ensured pathological relevance and safety, with no adverse effects observed during the 19-week intervention.

Regarding the pharmacodynamics of this living therapeutic, our current data establish that stable intestinal colonization (exceeding 10^5^ CFU) serves as an effective biological threshold sufficient to drive meaningful hepatic BAMBI translocation and subsequent fibrotic resolution. While this demonstrates a clear qualitative linkage between successful bacterial engraftment and therapeutic onset, we acknowledge that the precise quantitative relationship between graded colonization levels and the magnitude of therapeutic outcomes remains to be fully elucidated. This constitutes a limitation of the present study. Future investigations employing comprehensive dose-response analyses will be essential to define the optimal colonization dynamics required to maximize anti-fibrotic efficacy.

These findings pioneer an “engineered microbiome” framework for mechano-based therapy, integrating synthetic biology with pathophysiology. Future work should optimize bacterial colonization stability and BAMBI release kinetics for human translation, while addressing interspecies microbiome variability and long-term immunogenicity. Given the conserved role of TGF-β mechanotransduction in fibrosis, this platform may extend to other TGF-β-driven pathologies (e.g., renal/pulmonary fibrosis). Collectively, our study redefines anti-fibrotic strategies by prioritizing matrix modulation over mitogenic stimulation, offering a minimally invasive alternative for chronic liver disease management.

## 5 Conclusion

In conclusion, this study establishes a novel 'mechano-microbiome’ therapeutic paradigm for liver fibrosis. Unlike conventional small-molecule TGF-β inhibitors that often face systemic translational hurdles, or native probiotics that merely modulate general dysbiosis, our engineered Bs-BAMBI-8 platform provides a targeted, minimally invasive intervention. It uniquely distinguishes itself by actively disrupting the 'ECM stiffness → HSC activation’ mechanotransduction loop via the gut-liver axis. To advance clinical translation, future research must focus on specific, actionable parameters: (1) establishing precise quantitative dose-response models correlating intestinal bacterial loads with hepatic BAMBI concentrations; (2) elucidating the precise molecular pathways governing BAMBI’s traversal across the intestinal-vascular barrier, potentially utilizing high-resolution protein tracking to map the detailed transport kinetics of the gut-liver axis; (3) utilizing multi-omics profiling to decode secondary endogenous microbiome shifts induced by the intervention; and (4) engineering genetic biocontainment switches (e.g., auxotrophy) to ensure long-term environmental and immunological safety. Ultimately, this synthetic biology approach offers a robust and expandable blueprint for developing living medicines against a broad spectrum of mechanobiology-driven fibrotic diseases.

## Data Availability

The raw data supporting the conclusions of this article will be made available by the authors, without undue reservation.
