# Supplementary material for: Gut-engineered Bacillus subtilis-mediated BAMBI delivery for the treatment of thioacetamide-induced liver fibrosis through mechanotransduction inhibition
Source: Front Bioeng Biotechnol. 2026 May 8;14:1817519. doi: 10.3389/fbioe.2026.1817519 (PMC13193803; doi:10.3389/fbioe.2026.1817519)
Supplement: Supplementary file 1 [file Supplementaryfile1.docx]

Supplementary Material

# Supplementary Figures and Tables

## Supplementary Figures


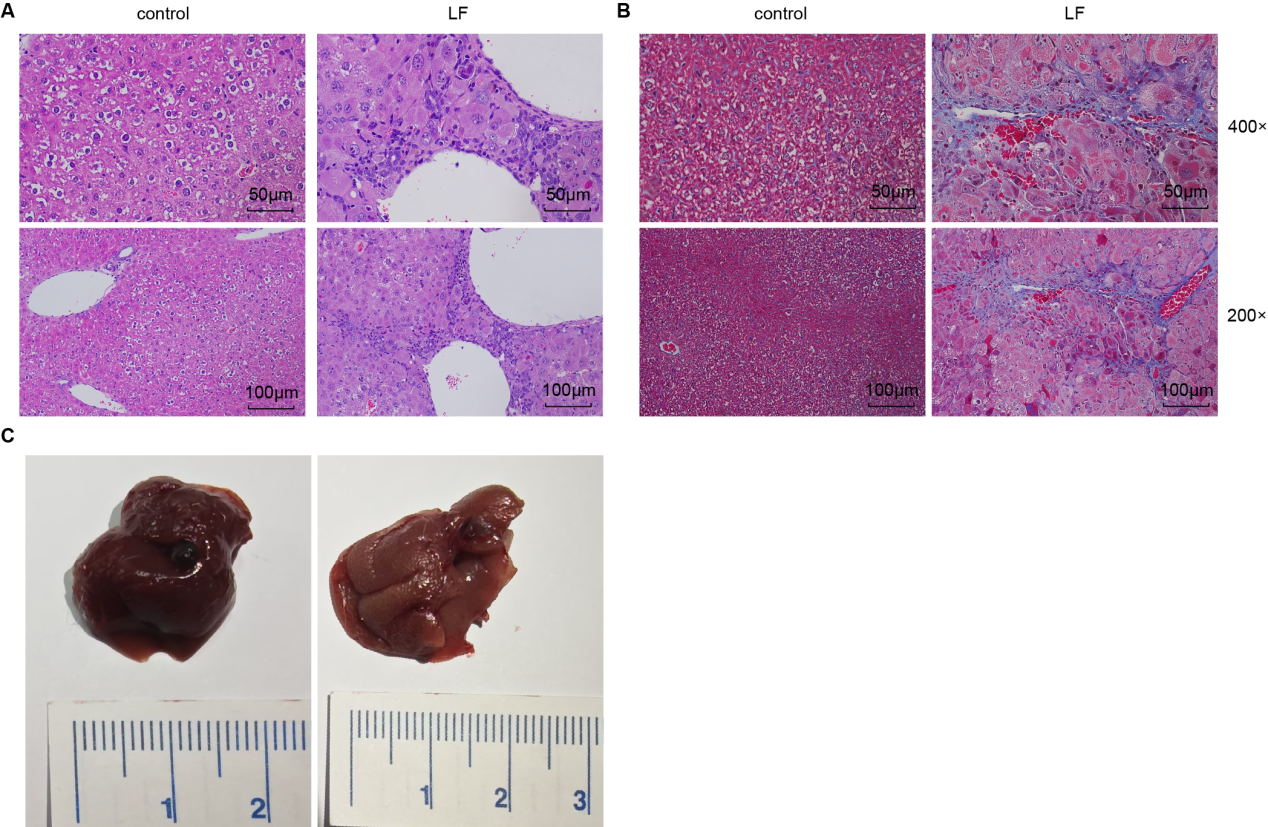


Fig.S1. Establishment of the mouse liver fibrosis model.

1. Representative hematoxylin and eosin (HE) stained liver sections showing significantly increased inflammatory infiltration in the fibrotic group compared to the control group. (B) Representative Masson's trichrome stained liver sections showing extensive collagen deposition (blue in Masson, typically representing fibrosis) in the fibrotic group, indicative of severe fibrosis, with minimal staining in the control group. (C) Gross appearance of liver specimens. The control liver displays a smooth and glossy surface, while the fibrotic liver exhibits a distinctly rough and dull surface.


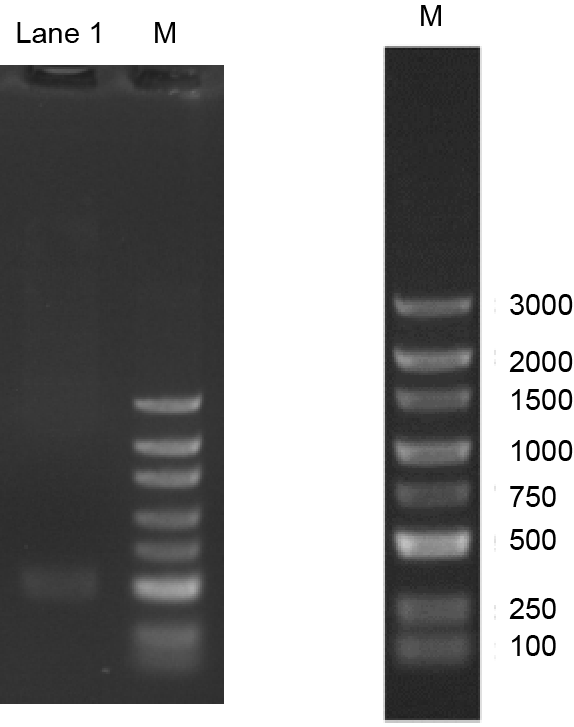


Fig. S2. PCR verification of the engineered Bacillus subtilis strain Bs-BAMBI-8.

Agarose gel electrophoresis of the colony PCR products to confirm the successful colonization of the engineered bacteria. Lane M represents the DNA molecular weight marker. Lane 1 shows the specific PCR amplicon obtained from the antibiotic-resistant colonies isolated from the intestinal contents, confirming the presence of Bs-BAMBI-8.


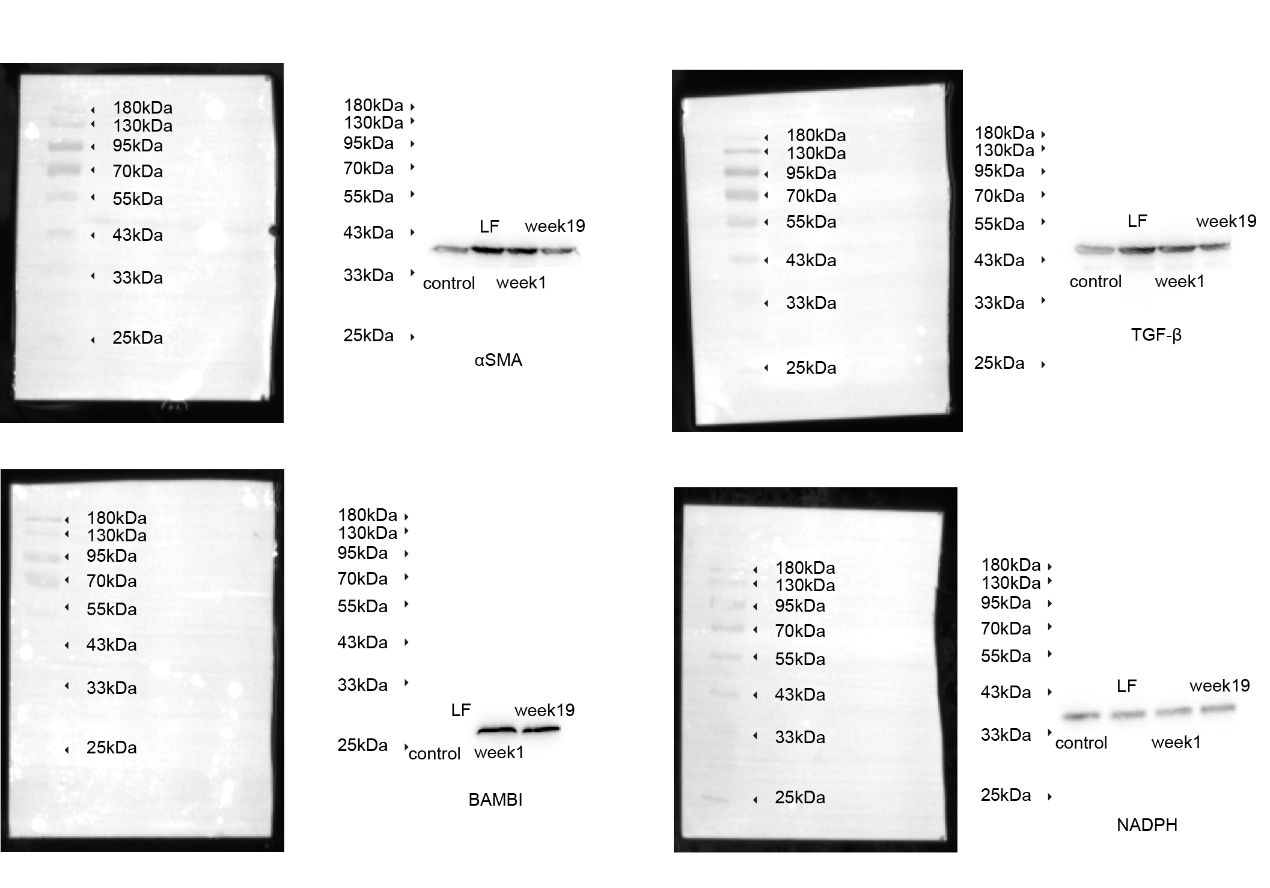


Fig.S3. The blots with molecular weight markers.

**
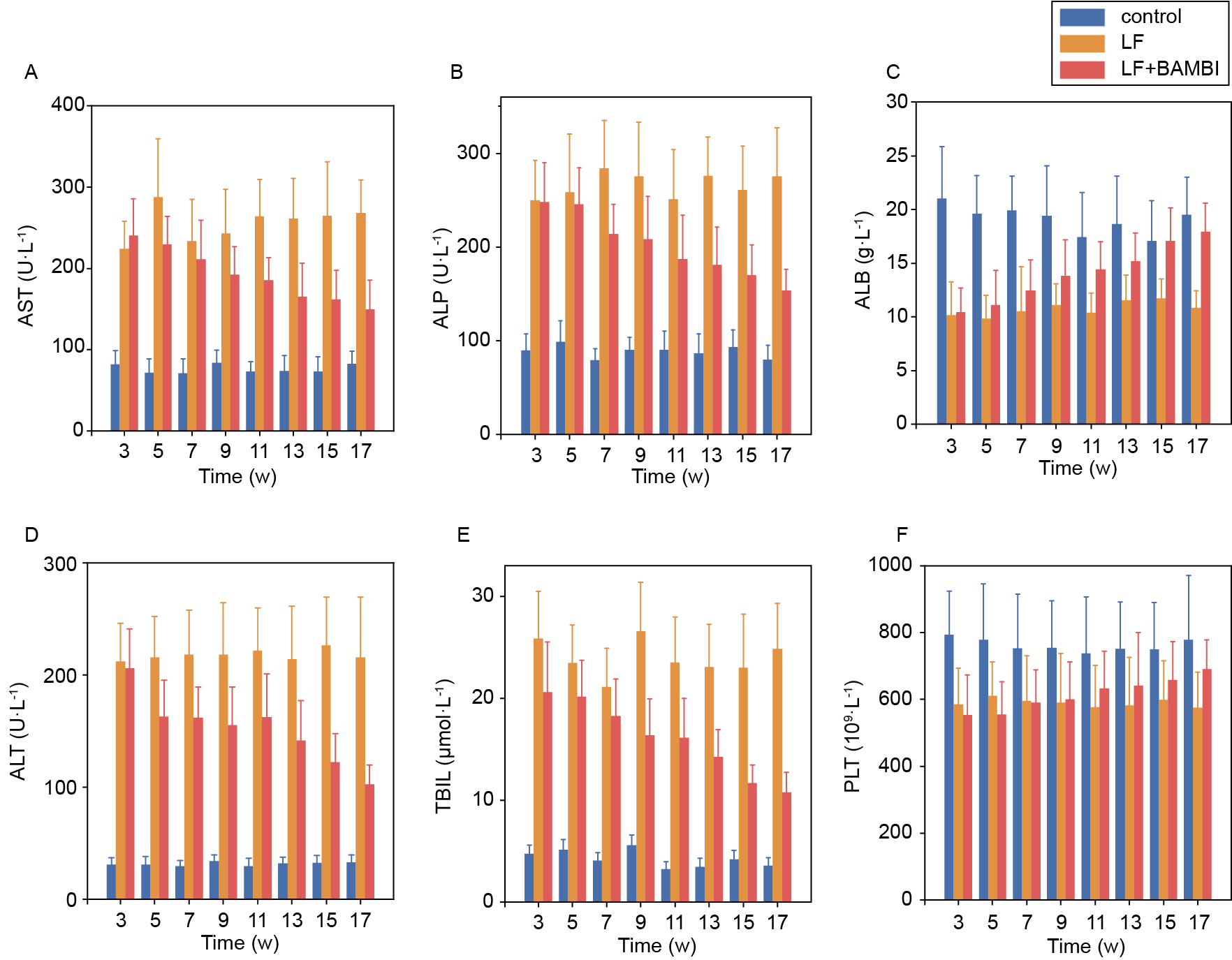
**

- Fig. S4. Detailed time-course analysis of serum biochemistry and hematological parameters across intermediate time points. (A) AST levels, (B) ALP levels, (C) ALB levels, (D) ALT levels, (E) TBIL levels, and (F) PLT counts were monitored every two weeks from week 3 to week 17. These intermediate data illustrate the continuous temporal dynamics of hepatic injury suppression and functional recovery in the LF+BAMBI group compared to the LF model group. Data are expressed as mean ± SD (n=5).
-
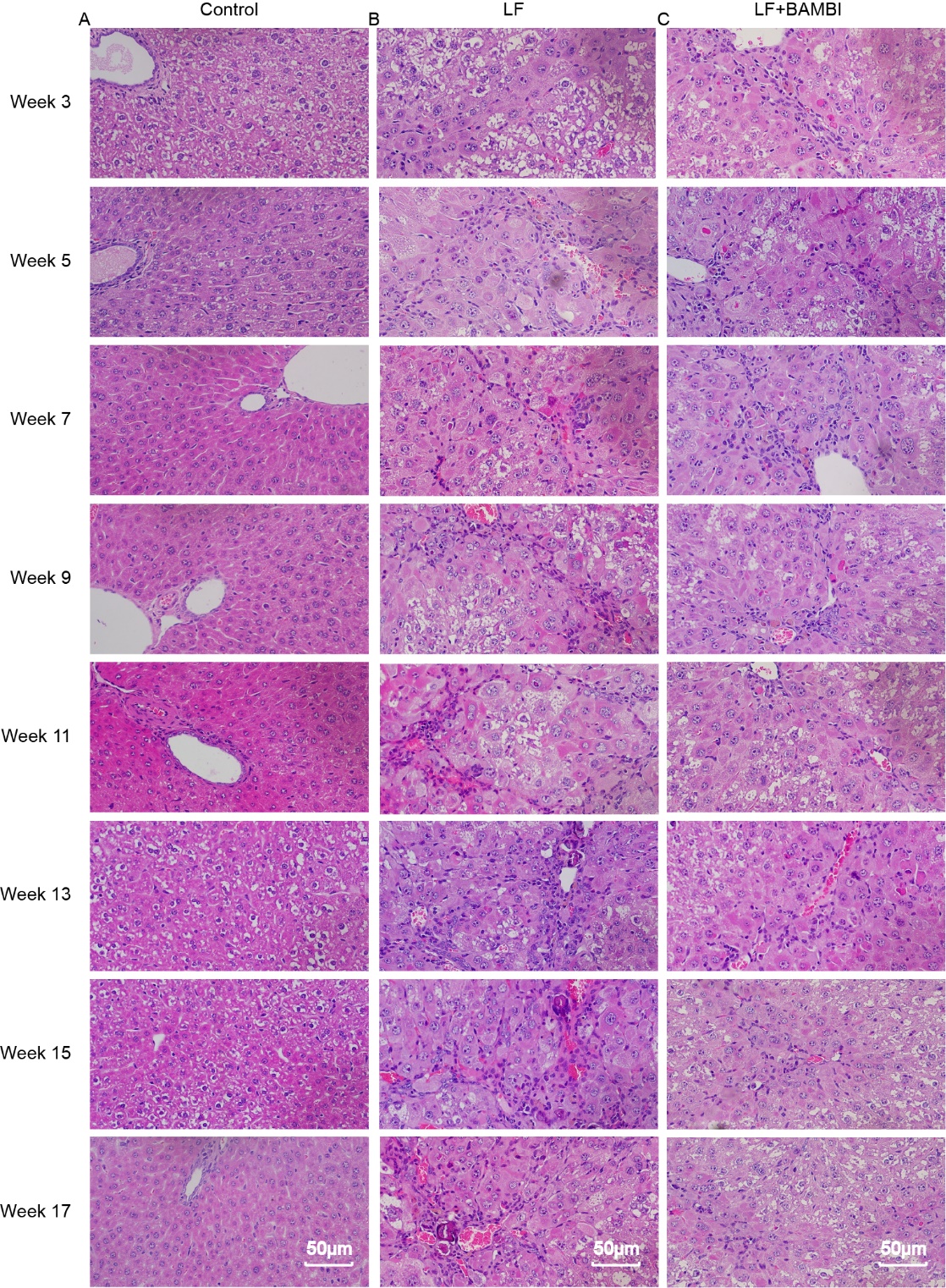

- Fig. S5. Detailed time-course of hepatic histopathological changes evaluated by HE staining. HE-stained liver sections from the (A) Control, (B) LF, and (C) LF+BAMBI groups at intermediate bi-weekly time points (weeks 3, 5, 7, 9, 11, 13, 15, and 17). Scale bars are 50 μm for all panels.
-
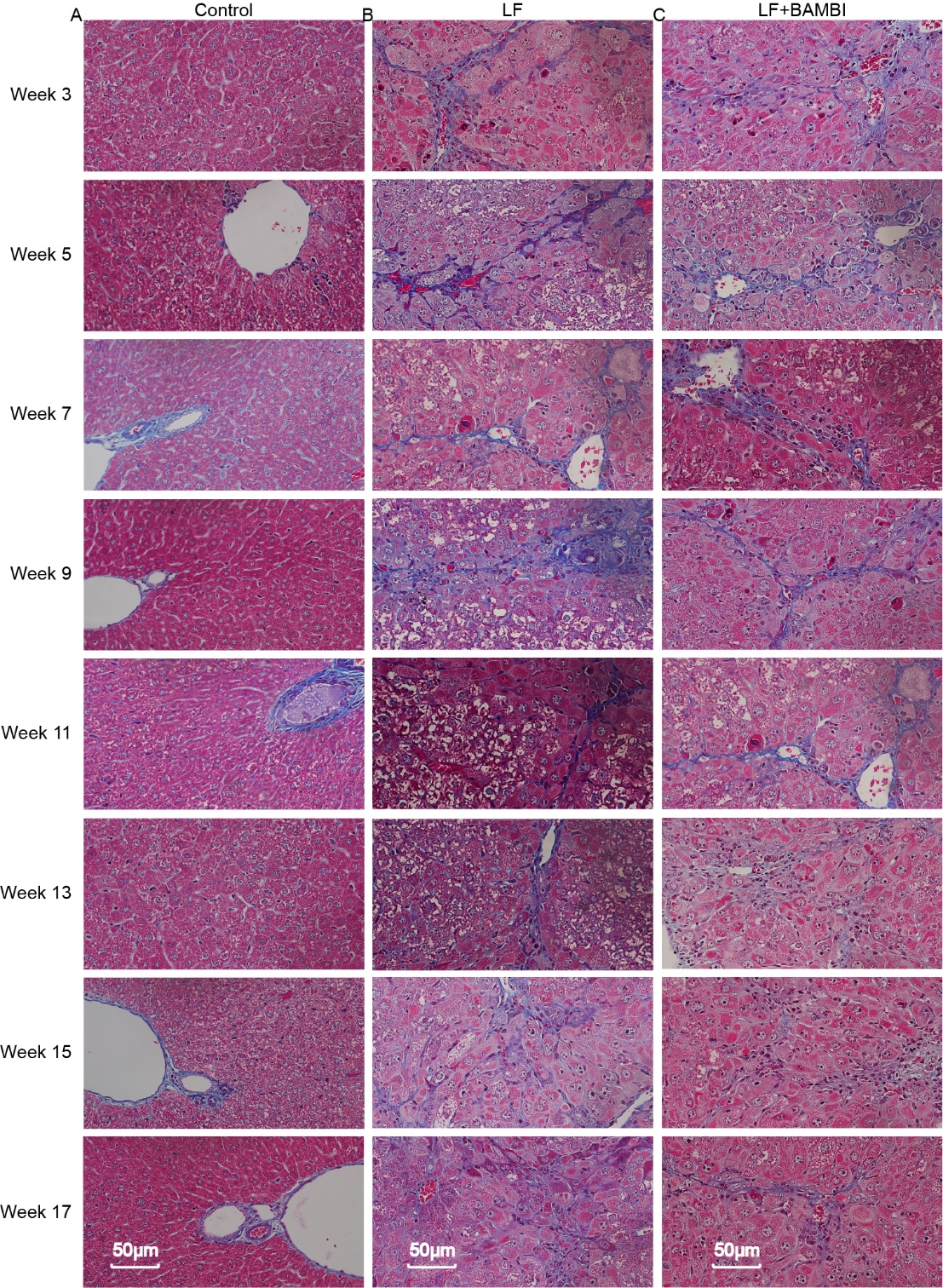

- Fig. S6. Detailed time-course of hepatic fibrotic changes evaluated by Masson's trichrome staining. Masson's trichrome-stained liver sections from the (A) Control, (B) LF, and (C) LF+BAMBI groups at intermediate bi-weekly time points (weeks 3, 5, 7, 9, 11, 13, 15, and 17). Scale bars are 50 μm for all panels.
-
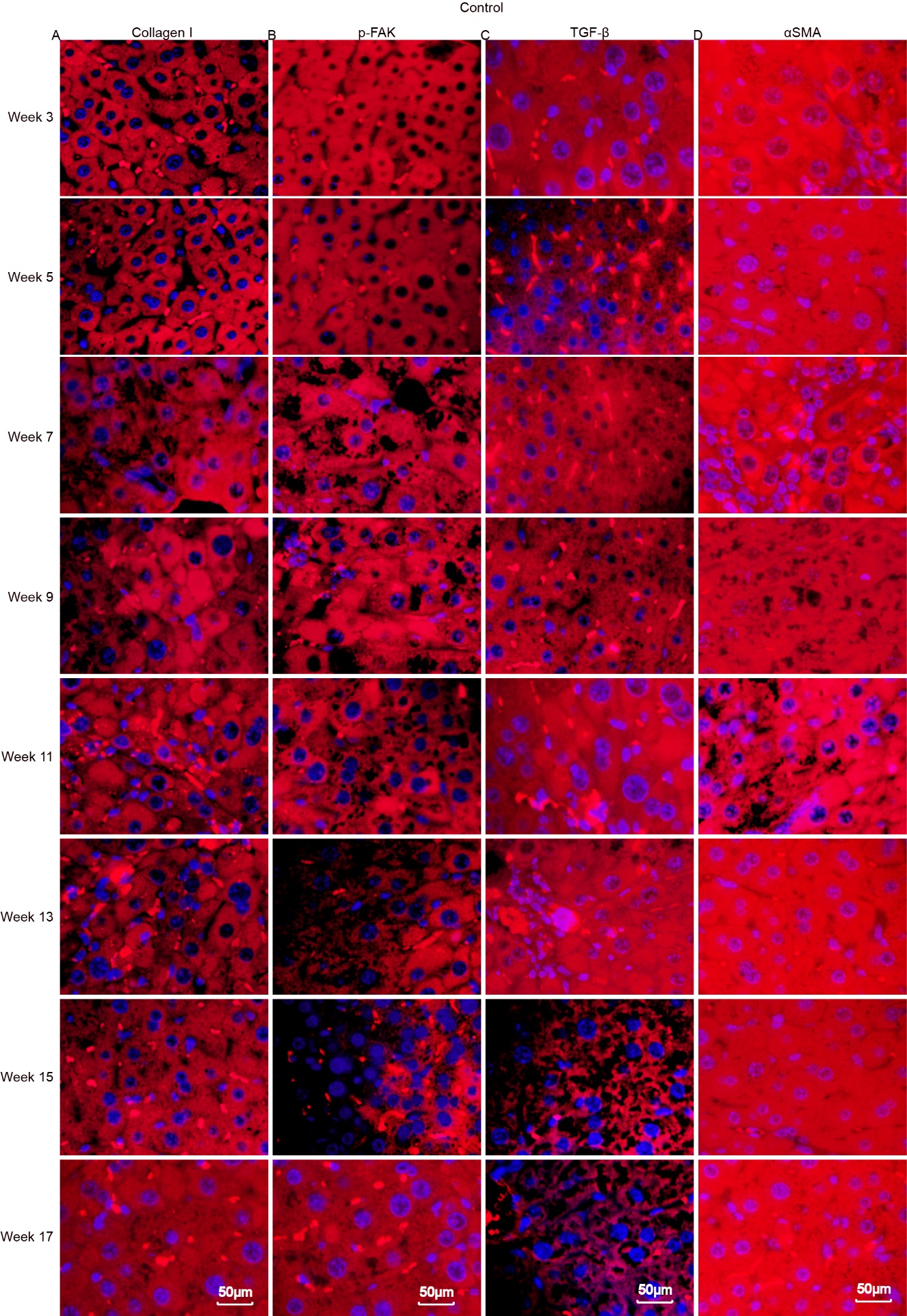

- Fig. S7. Detailed time-course of mechanotransduction markers in the Control group evaluated by immunofluorescence staining. Immunofluorescence images of liver sections from the Control group at intermediate bi-weekly time points (weeks 3, 5, 7, 9, 11, 13, 15, and 17). The panels display the expression of (A) Collagen I, (B) p-FAK, (C) TGF-β, and (D) α-SMA. Scale bars are 50 μm for all panels.
-
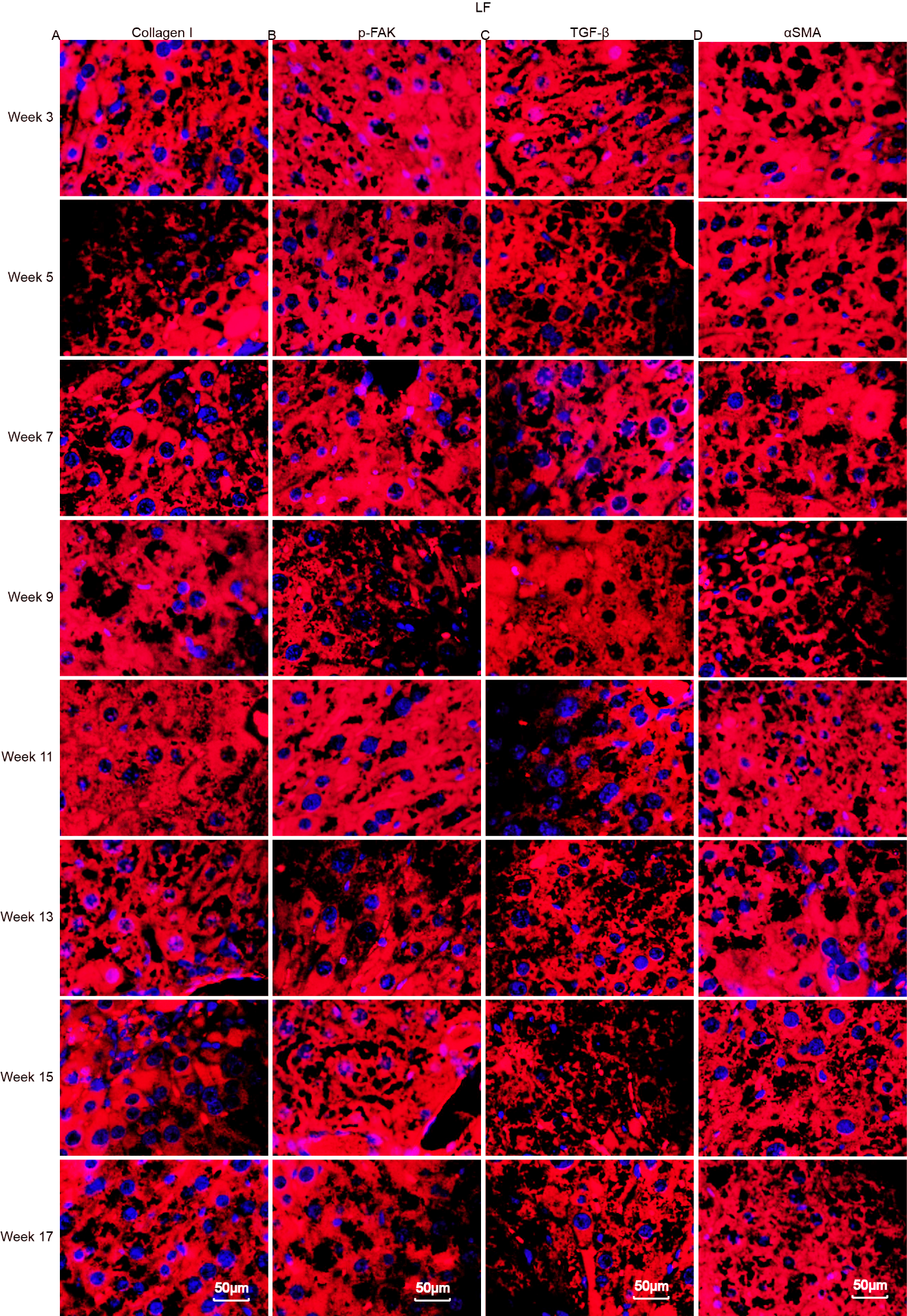

- Fig. S8. Detailed time-course of mechanotransduction markers in the LF group evaluated by immunofluorescence staining. Immunofluorescence images of liver sections from the LF group at intermediate bi-weekly time points (weeks 3, 5, 7, 9, 11, 13, 15, and 17). The panels display the expression of (A) Collagen I, (B) p-FAK, (C) TGF-β, and (D) α-SMA. Scale bars are 50 μm for all panels.
-
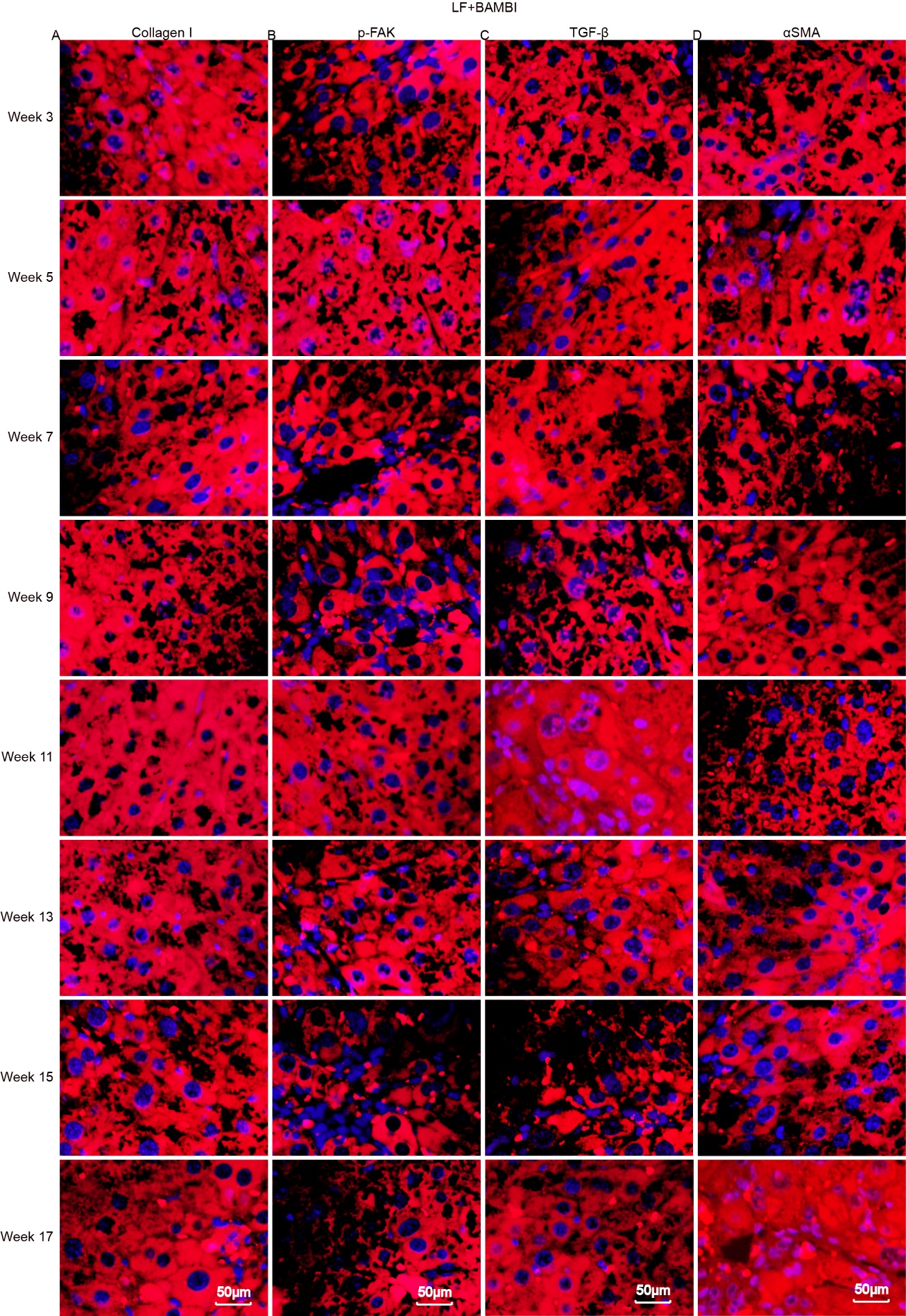

- Fig. S9. Detailed time-course of mechanotransduction markers in the LF+BAMBI group evaluated by immunofluorescence staining. Immunofluorescence images of liver sections from the LF+BAMBI group at intermediate bi-weekly time points (weeks 3, 5, 7, 9, 11, 13, 15, and 17). The panels display the expression of (A) Collagen I, (B) p-FAK, (C) TGF-β, and (D) α-SMA. Scale bars are 50 μm for all panels.

## Supplementary Tables

| Target | Sequence |
| --- | --- |
| BAMBI | GGTACCATGGACCGCCACTCTTCTTACATTTTTATTTGGCTCCAGCTTGAATTGTGCGCGATGGCTGTCTTGCTGACAAAAGGAGAAATCCGCTGCTATTGTGATGCCGCTCATTGTGTAGCCACTGGCTATATGTGTAAAAGCGAGCTGTCCGCTTGTTTTTCACGGCTCTTAGATCCGCAAAATTCAAACAGCCCTTTAACTCACGGCTGTCTTGATTCATTGGCAAGTACGACAGACATTTGCCAGGCAAAACAAGCAAGAAATCATTCAGGAACAACGATACCAACGCTGGAGTGCTGCCATGAAGATATGTGCAATTACCGAGGGCTGCATGATGTGCTTTCTCCCCCGCGTGGTGAAGCGTCGGGGCAAGGAAACCGTTATCAGCATGACGGCAGTAGGAACCTAATCACAAAGGTTCAAGAGCTTACCTCCAGCAAAGAATTATGGTTCAGAGCGAAGCTT |
| Primer 1(Forward) | TGGACCGCCACTCTTCTTAC |
| Primer 1(Reverse) | GCTTCGCTCTGAACCATAATTCTTT |

Table. S1 Sequence of BAMBI and specific primers used for the PCR verification of Bs-BAMBI-8.
